# Development and validation of an eco-compatible UV–Vis spectrophotometric method for the determination of Cu^2+^ in aqueous matrices

**DOI:** 10.1007/s00216-023-04785-6

**Published:** 2023-06-14

**Authors:** Prisco Prete, Davide Iannaccone, Antonio Proto, Marek Tobiszewski, Raffaele Cucciniello

**Affiliations:** 1 Environmental Chemistry Group (ECG), Department of Chemistry and Biology, University of Salerno, Via Giovanni Paolo II 132, 84084 Fisciano, SA Italy; 2 Department of Analytical Chemistry, Faculty of Chemistry and EcoTech Center, Gdańsk University of Technology (GUT), 11/12 G. Narutowicza St., 80-233 Gdańsk, Poland; 3 Centro Interdisciplinare Linceo Giovani, Accademia Nazionale dei Lincei, Via della Lungara, Roma, 10 - 00165, Italy

**Keywords:** Copper, AGREE, UV–vis spectrophotometry, Green analytical chemistry, Iminodisuccinic acid

## Abstract

Cu^2+^ are ubiquitous ions in the ecosystem and are responsible of serious environmental pollution. Indeed, the development of sensitive methods for Cu^2+^ detection is an urgent demand. In this work, we proposed a new spectrophotometric method for Cu^2+^ determination in different water matrices (distilled water, drinking water, wastewater, and river water). The method employs a bio-based organic ligand namely tetrasodium iminodisuccinate (IDS) able to form a stable complex with the analyte with a maximum absorption at 710 nm. Within the linear range of 6.3–381 mg L^−1^, the limit of detection (LOD) was determined to be as 1.43 mg L^−1^. Moreover, the recovery data of the spiked analysis of drinking/river/wastewater water samples were also satisfactory and verified the feasibility of the method for the analysis of Cu^2+^ in natural conditions. Finally, the AGREE assessment tool was used for a quantitative evaluation of the proposed method and reference method, in agreement with the green analytical chemistry principles. The results showed the lower environmental impact of the proposed method and the suitability of this novel approach for Cu^2+^ in water matrices.

**Graphical abstract:**

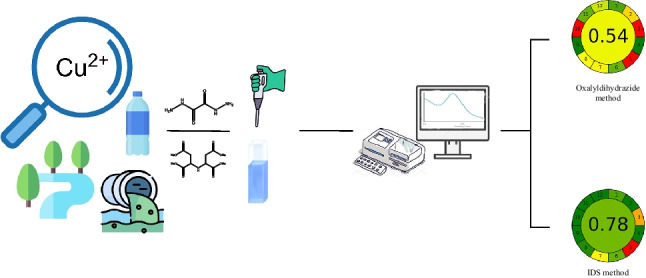

**Supplementary Information:**

The online version contains supplementary material available at 10.1007/s00216-023-04785-6.

## Introduction

Copper is a naturally occurring element present in all environmental media, including soil, sediment, and water. As essential metal, copper in traces is a micronutrient for cell function and plays a crucial role in different metabolic and enzymatic processes, as those involved in the antioxidant defense of the body and production of hemoglobin [1]. Metallic copper is widely used in electrical devices and widespread in the manufacturing of electrical cables and wires thanks to its high electrical conductivity and low price. Copper salts, including CuSO_4_, Cu(OH)_2_, and (Cu_3_Cl_2_(OH)_4_), are commonly used as fungicides and herbicides in viticulture [2]. As matter of facts, as an essential heavy metal, it is a micronutrient for cell function, whereas, depending on its concentration, Cu can damage environment and ecosystems, causing oxidative stress in plant cells [3], and it can be harmful for human bodies, causing severe diseases [4, 5]. To prevent environmental issues, several legislators worldwide imposed a concentration limit for Cu in drinking water, ground water, and wastewater. The World Health Organization (WHO) and the US Environmental Protection Agency (EPA) recommend the concentration of copper in drinking water not to exceed 31 µM and 20 µM, respectively [6, 7]. Italian legislation imposed in 2006 a concentration limit for the wastewater discharge in groundwater bodies to 1 mg L^−1^ [8]. The Italian Water Research Institute (IRSA) has proposed both an ICP-OES and a spectrophotometric analysis (based on the formation of a copper complex with oxalyldihydrazide) as reference methods for the determination of copper in aqueous matrices [9]. Notwithstanding spectrophotometric methods show higher values for LOD and LOQ in comparison with ICP-OES determination, they are a useful choice for the determination of copper in water matrices when ICP-OES sensitivity is not required. However, the application of oxalyldihydrazide-based method shows some limitations, due to the significant number of steps and harmful reagents, including mineral acids, acetaldehyde, and oxalyldihydrazide itself, required for the analysis. In this scenario, the development of several methods [10–15] for Cu^2+^ ion determination with satisfying sensitivity and selectivity has been proposed in literature, although characterized by some drawbacks. As a matter of fact, the determination of copper in food and beer matrices has been proposed using UV–Vis [14, 15] or F-AAS [10] spectroscopy coupled with a dispersive liquid–liquid microextraction (DLLME) preconcentration, which allowed to reach LOD values ranging from 10^−4^ to 10^−7^ mol L^−1^, but the DLLME implies the use of toxic CCl_4_ as extractant solvent. In addition, the analytical chemistry community attention to the development of new methods able to respect the green analytical chemistry (GAC) principles has gained in the last years [16–19]. Therefore, the development of an analytical method with high sensitivity for the determination of Cu^2+^ is of high interest as well as its adherence to GAC principles in comparison with the reference one [7]. Considering the development of a new spectrophotometric method, the choice of a bio-based ligand represents a promising option in the light of the 10th principle of GAC [20]. Recently, we investigated the use of tetrasodium iminodisuccinate (IDS) as ligand for Cu^2+^ in water solution. IDS can be easily obtained from bio-based maleic anhydride [21], and it is fully biodegradable [22, 23], in accordance with the green chemistry principles [24] and the benign by design approach [25].

The complex (Cu-IDS) formation was characterized by means of NMR spectroscopy, elemental analysis, and flame atomic adsorption spectroscopy (FAAS), and Cu-IDS has been used as active catalyst for the (photo-)Fenton process for water and wastewater treatment with encouraging results [26–29]. Furthermore, considering the high stability constant of Cu-IDS in distilled water (K_Cu-IDS_ = 10^13.1^) determined by potentiometric titrations by Hyvönen and co-workers [30], IDS is a valuable candidate for Cu^2+^ determination in water matrixes by spectrophotometry.

In this work, a new spectrophotometric method was developed with the aim to provide a rapid and simple strategy for the copper determination in different aqueous samples, with short time and low-cost analysis. This method relies on the formation of a stable, colorful complex of copper ion with IDS ligand. The Cu-IDS complex has been fully characterized, and it shows a significant absorbance peak at *λ* = 710 nm. Experiments were carried out in distilled water and then extended to real water samples (drinking water, urban wastewater, and river water). The role of interfering species such as other metal cations ubiquitous in an aqueous matrix (Mg^2+^ and Ca^2+^) and Fe^3+^, due to the high stability constant with IDS (K_Fe-IDS_ = 10^15.2^) was investigated. Furthermore, we evaluate and quantify the greenness of the new method according to recently developed AGREE assessment tool [31].

## Materials and methods

### Chemicals

Iminodisuccinate tetrasodium salt (Na_4_C_8_H_7_NO_8_, Na_4_IDS) (Baypure CX 100®) (CAS 144538–83-0) 65% wt was purchased from LANXESS (Cologna, Germany) and used without further purification. Copper sulfate pentahydrate (CuSO_4_ •5 H_2_O) (CAS 7758–99-8) ≥ 98.0% wt, calcium chloride (CaCl_2_) (CAS 10043–52-4) ≥ 93.0% wt, magnesium chloride hexahydrate (MgCl_2_ • 6 H_2_O) (CAS 7791–18-6) ≥ 93.0% wt, iron (III) chloride hexahydrate (FeCl_3_ • 6 H_2_O) (CAS 10025–77-1) 97% wt, oxalyldihydrazide (C_2_H_6_N_4_O_2_) (CAS 996–98-5) 98% wt, acetaldehyde (C_2_H_4_O) (CAS 75–07-0) ≥ 99.5% wt, and citric acid (C_6_H_8_O_7_) (CAS 77–92-9) ≥ 99.5% wt were purchased from Sigma-Aldrich (Saint Louis, MO, USA) and used without further purification.

### Standard solutions and instrumentation

Starting from commercial raw materials (Na_4_IDS 65% wt and CuSO_4_ •5 H_2_O 98% wt), an aqueous solution of Cu-IDS complex was prepared by dissolving stoichiometric amount of ligand and copper salt in distilled water [30]. As reported before, aqueous Cu-IDS complex was purified by column chromatography using silica gel as stationary phase and distilled water as mobile phase, to remove the impurities coming from the ligand production (35% wt due to maleate, fumarate, and aspartate sodium salts). The fractions containing the complex were collected and concentrated by using a rotary evaporator under reduced pressure. The as-obtained solid was dried at 120 °C overnight [26] and then characterized by means of ^13^C-NMR spectroscopy using a Bruker AVANCE 400 MHz NMR, elemental analysis with Thermo EA 1112 (CHNS/O) instrument and atomic absorption for the quantification of Cu and Na with a Perkin Elmer AAnalyst 100 Adsorption spectrophotometer. The high purity (> 99%) of the obtained Cu-IDS was confirmed.

A stock solution of Cu-IDS 4.6 mg mL^−1^ (15.0 mmol L^−1^) was prepared dissolving a proper amount of the purified complex in deionized water. Then, nine working standard solutions ranging from 0.10 to 6.0 mmol L^−1^ of Cu-IDS, corresponding to a range of copper concentrations from 6.3 to 381 mg L^−1^, were prepared by diluting the stock solution. The UV–visible absorbance spectra of these standard solutions were recorded by using a Cary Varian-50 spectrophotometer, and a calibration curve was built plotting the concentrations of copper in each standard solution versus the absorbance values at *λ* = 710 nm, in correspondence of the characteristic peak of Cu-IDS complex. In all cases, apart from the standard solutions, spectrophotometric analyses were carried out 5 min after adding the Na_4_IDS solution to Cu^2+^ containing samples.

### Method validation

The spectrophotometric method for quantification of copper in aqueous matrix through the formation of Cu-IDS complex was validated according to the International Conference on Harmonization (ICH) guidelines Q2 (R1) [32] in terms of linearity range, limit of detection (LOD), limit of quantification (LOQ), and selectivity.

The linearity range was determined by analyzing nine standard solutions with Cu-IDS concentrations in the range 0.10 mmol L^−1^–6.0 mmol L^−−1^ (Cu^2+^ from 6.3 to 381 mg L^−1^). The lowest concentration at which an analyte could be detected (LOD) and quantified (LOQ) were calculated as follow (Eq. (1) and Eq. (2)):

1 $$LOD=\frac{3.3\sigma }{S}$$

2 $$LOQ=\frac{10\sigma }{S}$$

where *S* is the slope and *σ* is the standard deviation of intercept of the calibration curve. Three replicates were analyzed for each sample, and the repeatability was evaluated to be 0.2% in all cases. Furthermore, selectivity tests have been performed to verify the interferences of other metal cations which may compete for the chelation sites of IDS with Cu^2+^. Ca^2+^, Mg^2+^, and Fe^3+^ have been chosen for the selectivity tests since they are ubiquitous metal cations, usually widespread within water matrices (K_Ca-IDS_ = 10^5.2^; K_Mg-IDS_ = 10^6.1^). Particularly, Fe^3+^ has been considered due to the high stability constant of Fe(III)-IDS (K_Fe-IDS_ = 10^15.2^) [33].

For this purpose, samples containing different concentrations of Cu^2+^, IDS, Ca^2+^, and Mg^2+^ or alternatively Fe^3+^ have been prepared. In detail, stock solutions of Cu^2+^ (1.0 g L^−1^, 15 mmol L^−1^), Na_4_IDS (3.37 g L^−1^, 10 mmol L^−1^), Ca^2+^ and Mg^2+^ (1.00 g L^−1^, 25.0 mmol L^−1^ Ca^2+^ and 41.6 mmol L^−1^ Mg^2+^), and Fe^3+^ (2.23 g L^−1^, 40 mmol L^−1^) have been prepared dissolving proper amounts of CuSO_4_, Baypure CX 100®, CaCl_2_, MgCl_2_ • 6 H_2_O and FeCl_3_ • 6 H_2_O, respectively, in distilled water.

Proper aliquots of these solutions have been diluted to prepare samples at different concentrations of Cu^2+^ (0.10–0.40 mmol L^−1^), IDS (0.10–1.2 mmol L^−1^) and the couple Ca^2+^ and Mg^2+^ (2.5 mmol L^−1^ and 4.16 mmol L^−1^, respectively) or Fe^3+^ (0.4–0.8 mmol L^−1^) before spectrophotometric analyses.

### Method applicability to real water matrices

To assess the applicability of this method to real aqueous samples, three different matrices have been investigated: a commercial drinking water, a sample collected from Irno river (40.673382 14.773848, Salerno, Southern Italy), and one collected from a wastewater treatment plant (Nocera Inferiore, Southern Italy). The concentration of copper in these samples has been previously determined by means of ICP-OES. Since the concentration of the analyte was found to be neglectable (< 0.04 mg L^−1^ for each sample), the standard spiking method was used to obtain samples with known concentration of copper (0.4 mmol L^−1^). Subsequently, copper concentrations were determined using the new spectrophotometric method after the addition of equimolar amount of IDS and by means of ICP-OES as reference. Data were compared to evaluate the applicability of the newly developed method to different water matrixes.

### Greenness evaluation using AGREE

The agreement of the investigated methods with the 12 principles of green analytical chemistry (GAC) was discussed using the software AGREE [31]. In detail, the spectrophotometric method developed in this work was compared to the spectrophotometric method for the determination of copper which involves the use of oxalyldihydrazide.

For each principle, AGREE evaluates the performance of the proposed analytical method by assigning a score. The scores are pre-defined by a well-defined model ranging from 0 to 1 depending on the accordance of the method with the principle. The score depends on the estimation of the input parameters to insert in AGREE software.

Equal weights have been set for all 12 principles, thus assuming that each principle of GAC is equally important for the AGREE analysis. The input parameters and the respective output scores for both methods have been listed in Table S2 (see Supporting Information).

## Results and discussion

### Method validation

#### Calibration, LOD, and LOQ

The calibration curve was obtained using data of the spectrophotometric analysis of nine standard solutions containing Cu-IDS (λ_max_ = 710 nm) in the concentrations range 0.1–6.0 mmol L^−1^, corresponding to [Cu^2+^] in the range 6.3–381 mg L^−1^.

**Fig. 1:**
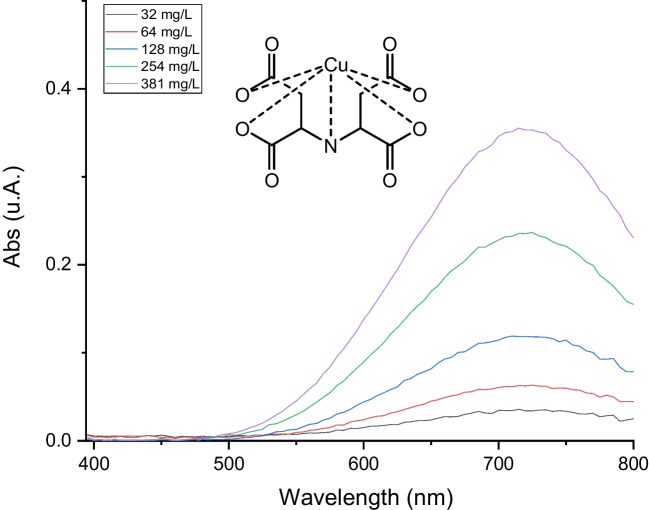
Absorbance spectra of Cu-IDS at different Cu^2+^ concentrations. Inset: Structure of Cu-IDS complex

The reaction between Cu^2+^ and Na_4_IDS is described in Eq. (3), and the recorded spectra have been reported in Fig. 1. Also, inset of Fig. 1 shows the structure of Cu-IDS complex.

3 $${\mathrm{Cu}}^{2+}+{\mathrm{IDS}}^{4-}\to {\mathrm{Cu}-\mathrm{IDS}}^{2-}$$

**Fig. 2:**
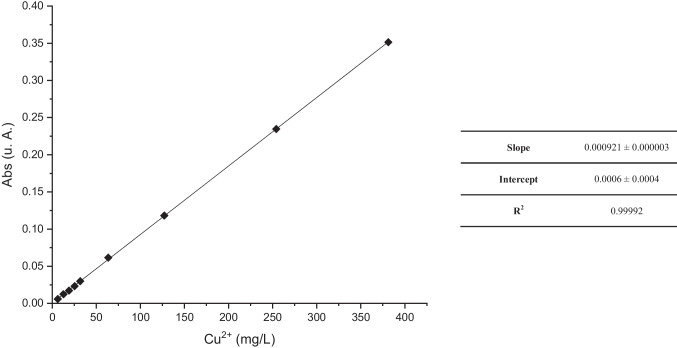
Calibration curve and regression data for spectrophotometric determination of Cu^2+^ via spectrophotometric quantification of Cu-IDS complex (number of repetitions = 3)

Coefficients of the calibration curve were calculated by the least squares method and reported below in Fig. 2. The obtained value of *R*^2^ = 0.99992 clearly states the linearity of the developed method over the range under investigation.

LOD and LOQ values were calculated using Eqs. (1) and (2), respectively, from the linear regression data of the calibration curve reported in Fig. 2, and the results were, respectively, of LOD = 1.43 mg L^−1^ and LOQ = 4.34 mg L^−1^. The Cu-IDS complex shows high stability in an extended pH range (2–8) allowing the application of the proposed method for the determination of Cu^2+^ in different water matrices.

#### Selectivity tests

The interferences of Ca^2+^ and Mg^2+^ on the determination of Cu^2+^ has been evaluated by spectrophotometry of samples containing increasing concentrations of Cu^2+^ and IDS (1:1 molar ratio) in the presence of an excess of calcium and magnesium ions (100 mg L^−1^). The concentration of Cu^2+^ determined by spectrophotometry was compared to the concentration of copper added to each sample (Table 2). The latter allows to verify the interferences of other cations.

As shown in Table 1, the significant accordance between the experimental and theoretical copper concentration allowed to assess that calcium and magnesium does not interfere in the determination of copper with this method, even when the analyte concentration was close to the LOQ (0.07 mmol L^−1^).

**Table 1:** Interference test of Ca^2+^ and Mg^2+^ cations in spectrophotometric determination of Cu^2+^ as Cu-IDS in presence of Ca^2+^ 2.5 mmol L^−1^ and Mg^2+^ 4.16 mmol L^−1^ (100 mg L^−1^ each) (number of repetitions = 3)

[Cu^2+^] added (mmol L^−1^)	[Cu^2+^] measured (mmol L^−1^)
0.10	(0.104 ± 0.008)
0.20	(0.189 ± 0.008)
0.30	(0.312 ± 0.009)
0.40	(0.411 ± 0.009)

Considering the higher stability constants for Cu^2+^/Fe^3+^-IDS, ad hoc experiments were carried out in the presence of both stoichiometric and excess molar concentrations of Fe^3+^ compared to Cu^2+^. In this case, the Cu-IDS concentration has been determined in solutions containing Cu^2+^ 0.4 mmol L^−1^, Fe^3+^ and IDS at different molar ratios (Table 2).

**Table 2:** Interference tests of Fe^3+^ in the spectrophotometric determination of Cu^2+^ (number of repetitions = 3)

	[Cu^2+^]/[Fe^3+^]/[IDS] (mmol L^−1^)	Molar ratios Cu/Fe/IDS	[Cu^2+^] measured (mmol L^−1^)
1	0.40/0.40/0.40	1/1/1	(0.275 ± 0.009)
2	0.40/0.40/0.80	1/1/2	(0.404 ± 0.009)
3	0.40/0.80/1.2	1/2/3	(0.389 ± 0.009)

In this case, as shown in entry 1 of Table 2, ferric ion does interfere with the Cu measurement due to the high stability constant K_1_ for the formation of the complex [Fe-IDS]^−^, which is higher than the stability constant K_2_ of the complex with copper.

$$\begin{array}{cc}{Fe}^{3+}+IDS\rightleftharpoons Fe-IDS& \mathrm{log }{\mathrm{K}}_{1} = 15.2\end{array}$$

$$\begin{array}{cc}{Cu}^{2+}+IDS\rightleftharpoons Cu-IDS& \mathrm{log }{\mathrm{K}}_{2}= 13.1\end{array}$$

In fact, the copper concentration determined by spectrophotometry reaches 70% compared to the theoretical one. However, this interference can be removed by adding a proper amount of ligand in order to chelate both iron and copper ions in solution*.* In fact, as highlighted in entries 2–3 in Table 2, the use of a proper amount of ligand inhibits the interference of Fe^3+^ also in the case of large excess of iron with respect to copper.

#### Matrix effect evaluation

Three different water samples (bottled water, river water, and urban wastewater) have been collected and analyzed using a Perkin Elmer Optima 7000 DV ICP-OES to measure the initial concentration of copper. Since it was neglectable (< 0.04 mg L^−1^ for each sample), copper sulfate was added to reach a Cu^2+^ concentration of 0.4 mmol L^−1^. Then, to assess whether the matrix effect could affect the quantitative determination of copper with the spectrophotometric method, equimolar amount of IDS was added to the samples prior to the spectrophotometric analyses (see data in Table 3).

**Table 3:** Effect of real matrices on the spectrophotometric determination of copper as Cu-IDS (number of repetitions = 3)

Sample	[Cu^2+^] added (mmol L^−1^)	[Cu^2+^] measured (mmol L^−1^)
Drinking water	0.40	(0.372 ± 0.009)
River water	0.40	(0.413 ± 0.009)
Urban wastewater	0.40	(0.389 ± 0.009)

Data reported in Table 3 clearly highlight that the developed method is applicable to different real matrices. In fact, different matrices under investigation do not affect the determination of Cu^2+^, meaning that none of their components may interfere with the chelation reaction, mask the analyte, or give (positive or negative) interference during the spectrophotometric measurement. The obtained recovery of 97–103% highlights the applicability of the IDS-based method. Data are in line with those reported for the oxalyldihydrazide-based method, ranging from 86 to 105% (LOD = 0.053 mg L^−1^ and LOQ = 0.16 mg L^−1^). In conclusion, the proposed protocol for real water samples includes the use of 9 mL of sample which will be treated with 1 mL of a 0.30 mol L^−1^ solution of Na_4_IDS in the presence of samples with [Cu^2+^] = 6.0 mmol L^−1^ and [Fe^3+^] = 12.0 mmol L^−1^ (dilution of the water sample will be needed at higher Cu^2+^ and Fe^3+^ concentrations). Then, spectrophotometric analyses are carried out 5 min after adding the Na_4_IDS solution. At the best of our knowledge, the proposed methodology is also economically accessible due to the commercial price of IDS (10 euro/kg).

#### AGREE

**Fig. 3:**
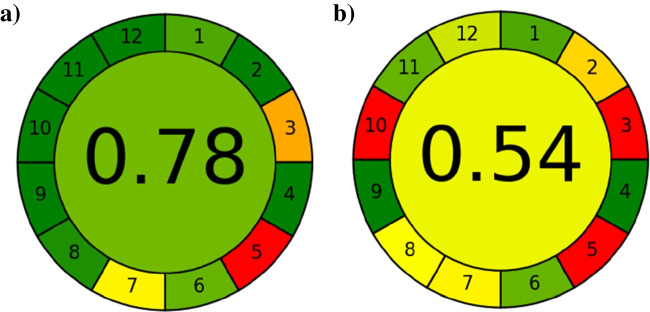
Comparison of output graphical results of AGREE analysis for **a** IDS-based method and **b** oxalyl hydrazide-based method

The developed method was compared to the oxalyl hydrazide-based spectrophotometric method for the determination of copper on the basis of their accordance with the 12 principles of green analytical chemistry using the AGREE software. Results are summarized in Fig. 3.

The final score of the AGREE evaluation highlights the higher greenness obtained by the proposed spectrophotometric method. This is mainly a result of a faster and simple derivatization of the sample before the analysis, through the formation of the Cu-IDS complex, compared to the oxalyldihydrazide method. This advantage results in an appreciable improvement of the score associated to the 8th principle. The substitution of the oxalyldihydrazide with the eco-friendly and biomass-derived iminodisuccinate ligand used for the determination of copper also affects other principles (10th, 11th, and 12th). The worse scores associated to the 3rd, 5th, and 7th principles, rely on the spectrophotometric determination of the analyte.

## Conclusions

Iminodisuccinate tetrasodium salt (Na_4_IDS) is a biomass-derived and biodegradable ligand which has been exploited in this work to develop a new spectrophotometric method for the determination of copper in aqueous matrices. The method shows linearity in the concentrations range 6.3 mg L^−1^–381 mg L^−1^ with LOD and LOQ values of 1.43 mg L^−1^ and 4.34 mg L^−1^, respectively. This work demonstrated the selectivity of the proposed method toward Cu^2+^ also in presence of high concentrations of Ca^2+^, Mg^2+^, and Fe^3+^. The positive interference of Fe^3+^ can be easily suppressed by adding an excess of ligand.

Moreover, also the matrix effect has been proved to be neglectable on the spectrophotometric determination of the analyte, since the recovery of copper added to three different aqueous matrices (drinking water, river water, and urban wastewater) was found to be quantitative. Finally, the proposed method and the reference spectrophotometric method with oxalyldihydrazide have been compared according to the 12 principles of GAC using the software AGREE. Results show higher greenness obtained by the proposed spectrophotometric method compared to the reference one.

## Supplementary Information

Below is the link to the electronic supplementary material.

Supplementary file1 (DOCX 30 KB)
