# Supplementary material for: Development and validation of an eco-compatible UV–Vis spectrophotometric method for the determination of Cu2+ in aqueous matrices
Source: Anal Bioanal Chem. 2023 Jun 14;415(20):5003–10. doi: 10.1007/s00216-023-04785-6 (PMC10386959; doi:10.1007/s00216-023-04785-6)
Supplement: Supplementary file 1 — Supplementary file1 (DOCX 30 KB) [file 216_2023_4785_MOESM1_ESM.docx]

**SUPPORTING MATERIALS**

**Development and validation of an eco-compatible UV-Vis spectrophotometric method for the determination of Cu^2+^ in aqueous matrices**

Prisco Prete,^a^ Davide Iannaccone, ^a^ Antonio Proto,^a^ Marek Tobiszewski,^b^ Raffaele Cucciniello ^a^*

^a^ Environmental Chemistry Group (ECG), Department of Chemistry and Biology, University of Salerno, via Giovanni Paolo II 132, 84084 Fisciano (SA), Italy

^b^ Department of Analytical Chemistry, Faculty of Chemistry and EcoTech center, Gdańsk University of Technology (GUT), 11/12 G. Narutowicza St., Gdańsk 80-233, Poland

*Corresponding author: Dr. Raffaele Cucciniello, ^a^ University of Salerno via Giovanni Paolo II,132- 84084 Fisciano (SA) tel +39 89969366, e-mail: [rcucciniello@unisa.it](mailto:rcucciniello@unisa.it)

**Table S1. Chemical characterization of the investigated water matrices.**

| **Parameter** | **Drinking water** | **Wastewater** | **Irno river** |
| --- | --- | --- | --- |
| pH | 6,9 | 7,5 | 7,0 |
| Ca^2+^ (mg L^-1^) | 86,2 | 65,6 | 25,8 |
| Mg^2+^ (mg L^-1^) | 12,0 | 33,7 | 57,6 |
| Na^+^ (mg L^-1^) | 3,4 | 221,6 | 22,2 |
| Cl^-^ (mg L^-1^) | 5,3 | 315,3 | 107,6 |
| K^+^ (mg L^-1^) | 1,0 | 27,8 | 29,0 |
| NO_3_^-^ (mg L^-1^) | 3,1 | 8,6 | 653,3 |
| F^-^ (mg L^-1^) | 0,1 | 0,2 | 0,1 |
|  |  |  |  |

| **Slope** | 0.494 ± 0.008 |
| --- | --- |
| **Intercept** | 0.035 ± 0.008 |
| **R^2^** | 0.99 |

**Figure S1. Calibration curve for spectrophotometric determination of Cu via Oxalyldihydrazide based-method**

**LOD =** 0.053 mg/L **LOQ =** 0.16 mg/L

**Table S2.** *Input parameters and output scores for AGREE analysis of the Cu using the new proposed method with IDS and the oxalyldihydrazide-based spectrophotometric method.*

|  | **Input** | | **Output** | |
| --- | --- | --- | --- | --- |
|  | **Cu-IDS** | **Oxalyldihydrazide** | **Cu-IDS** | **Oxalyldihydrazide** |
| 1 | In-field sampling and direct analysis | In-field sampling and direct analysis | 0.85 | 0.85 |
| 2 | 5 mL sample | 5 mL sample | 0.42 | 0.42 |
| 3 | At line analysis | Off line analysis | 0.33 | 0.0 |
| 4 | 3 or fewer steps required | 3 or fewer steps required | 1.0 | 1.0 |
| 5 | Manual and not miniaturized | Manual and not miniaturized | 0.0 | 0.0 |
| 6 | Derivatization with non-hazardous (IDS) | Derivatization with non-hazardous (Oxalyldihydrazide) | 0.8 | 0.8 |
| 7 | 5 mL waste | 5 mL waste | 0.48 | 0.48 |
| 8 | 1 analyte per run; 60 measures/h | 1 analyte per run; 10 measures/h | 0.94 | 0.51 |
| 9 | UV-Vis spectrophotometer | UV-Vis spectrophotometer | 1.0 | 1.0 |
| 10 | All reagents are bio-based | None of the reagents are from bio-based sources | 1.0 | 0.0 |
| 11 | No toxic reagents | Toxic reagent used  (0.5 mL acetaldehyde) | 1.0 | 0.59 |
| 12 | No hazard | Toxic to aquatic life  Explosive | 1.0 | 0.6 |
